# *Alternaria* host-specific (HSTs) toxins: An overview of chemical characterization, target sites, regulation and their toxic effects

**DOI:** 10.1016/j.toxrep.2019.06.021

**Published:** 2019-07-17

**Authors:** Mukesh Meena, Swarnmala Samal

**Affiliations:** a Department of Botany, University College of Science, Mohanlal Sukhadia University, Udaipur, 313001, India; b Centre of Advanced Study in Botany, Institute of Science, Banaras Hindu University, Varanasi, 221005, India

**Keywords:** SMs, secondary metabolites, HSTs, host specific toxins, nHSTs, non-host specific toxins, TEN, tentoxin, AOH, alternariol, AME, alternariol 9-monomethyl ether, ALT, alternuene, TeA, tenuazonic acid, ATX, alterotoxin, DHT, dihydrotentoxin, PKS, polyketide synthase gene, PCD, programmed cell death, NO, nitric oxide, ROS, reactive oxygen species, HR, hypersensitive response, H_2_O_2_, hydrogen peroxide, ^1^O_2_, singlet oxygen, O_2_**˙**ˉ, superoxide anion, **˙**OH, hydroxyl radical, SOD, superoxide dismutase, GPX, guaiacol peroxidase, CAT, catalase, GR, glutathione reductase, APX, ascorbate peroxidase, MDHAR, monodehydroascorbate reductase, DHAR, dehydroascorbate reductase, GSH, glutathione, AA, ascorbic acid, CDCs, conditionally dispensable chromosomes, REMI, restriction enzyme-mediated integration, NRPS, nonribosomal peptide synthetase, UGT, UDP-Glucuronosyltransferases, *Alternaria* species, Secondary metabolites, Pathogenicity, Host-specific toxins, Non-host-specific toxins

## Abstract

*Alternaria* causes pathogenic disease on various economically important crops having saprophytic to endophytic lifecycle. Pathogenic fungi of *Alternaria* species produce many primary and secondary metabolites (SMs). *Alternaria* species produce more than 70 mycotoxins. Several species of *Alternaria* produce various phytotoxins that are host-specific (HSTs) and non-host-specific (nHSTs). These toxins have various negative impacts on cell organelles including chloroplast, mitochondria, plasma membrane, nucleus, Golgi bodies, etc. Non-host-specific toxins such as tentoxin (TEN), Alternaric acid, alternariol (AOH), alternariol 9-monomethyl ether (AME), brefeldin A (dehydro-), Alternuene (ALT), Altertoxin-I, Altertoxin-II, Altertoxin-III, zinniol, tenuazonic acid (TeA), curvularin and alterotoxin (ATX) I, II, III are known toxins produced by *Alternaria* species. In other hand, *Alternaria* species produce numerous HSTs such as AK-, AF-, ACT-, AM-, AAL- and ACR-toxin, maculosin, destruxin A, B, etc. are host-specific and classified into different family groups. These mycotoxins are low molecular weight secondary metabolites with various chemical structures. All the HSTs have different mode of actions, biochemical reactions, and signaling mechanisms to causes diseases in the host plants. These HSTs have devastating effects on host plant tissues by affecting biochemical and genetic modifications. Host-specific mycotoxins such as AK-toxin, AF-toxin, and AC-toxin have the devastating effect on plants which causes DNA breakage, cytotoxic, apoptotic cell death, interrupting plant physiology by mitochondrial oxidative phosphorylation and affect membrane permeability. This article will elucidate an understanding of the disease mechanism caused by several *Alternaria* HSTs on host plants and also the pathways of the toxins and how they caused disease in plants.

## Highlights

• *Alternaria* is responsible to cause pathogenic diseases on several crops.
• *Alternaria* species produce several types of host-specific and non-host-specific toxins.
• HSTs have devastating effects on host plants by affecting biochemical and genetic alterations.
• Article will provide an idea to understand the disease mechanism caused by HSTs on hosts.

## Graphical abstract

**Figure:**
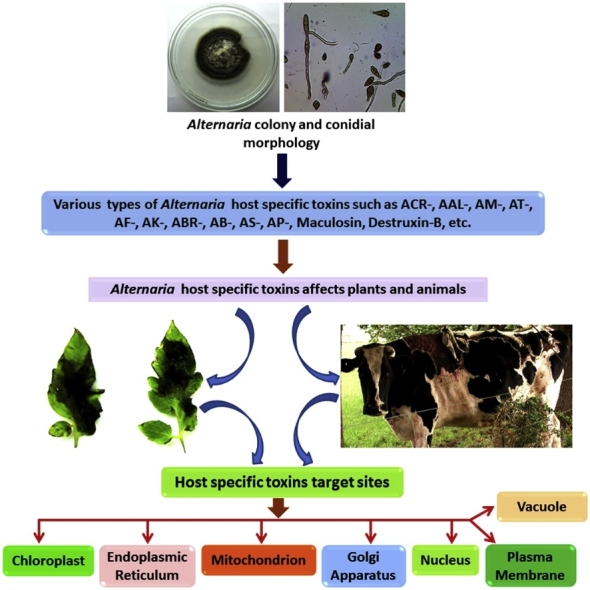

## 1 Introduction

The genus *Alternaria* is ubiqutenious in nature, imperfecti fungi that belong to the phylum Ascomycetes of the Hyphomycetes [1,2]. It contains both saprophytic and endophytic in nature which is causal agents of various crops, fruits, and vegetable diseases. Till date, nearly 300 species of *Alternaria* have been reported [3]. These include *Alternaria alternata*, *Alternaria arborescens*, *Alternaria radicina*, *Alternaria brassicola*, *Alternaria brassicae*, and *Alternaria infectoria* [1,[3], [4], [5]]. *A. alternata* causes disease in various economically important plants like brochelli, tomato, chili, potato, citrus, apple, etc. [6]. In 1933, the first black rot disease caused by *Alternaria* on Japanese pear was reported [7,8]. Various secondary metabolites produced by *Alternaria* species those maybe host-specific and non-host-specific toxins at the different stage of pathogenesis [9,10,6,11,12].

More than 70 toxins have been reported to be produced by *Alternaria* fungal pathogenicity species [[13], [14], [15], [16], [17], [18]]. In *Alternaria* 20 HSTs has been reported [1,[19], [20], [21], [22], [23], [24], [25],^4^]). Host-specific toxins with low molecular weight are common in seven *Alternaria* species and four *Cochliobolus* species [26]. *A. alternata* HST produces various pathotypes in structure [[26], [27], [28]].

On the basis of chemical structure *Alternaria* mycotoxin is divided into five classes; (1) dibenzopyrone derivatives, which encompass alternariol (AOH), alternariol monomethyl ether (AME), and altenuene (ALT); (2) tetramic acid derivatives, comprise tenuazonic acid (TeA), and *iso*-tenuazonic acid (*iso*-TeA); (3) perylene derived, altertoxins I, II, and III (ATX-I, ATX-II, and ATX-III); (4) *A. alternata* f. sp. *lycopersici* TA1, TA2, TB1, and TB2 toxin (AAL TA1, TA2, TB1, and TB2); (5) wide-ranging structures, such as tentoxin (TEN), *iso*-tentoxin (*iso*-TEN), and dihydrotentoxin (DHT), which are cyclic tetrapeptide [[29], [30], [31], [32], [33], [34]].

**Fig. 1:**
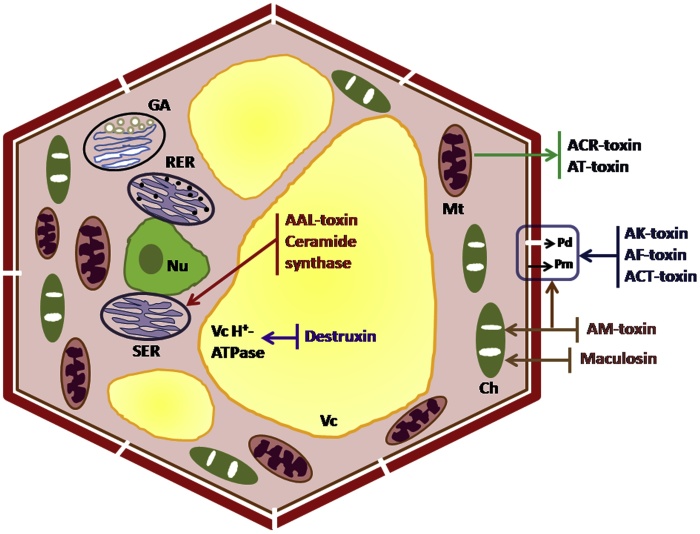
Schematic presentation of target sites of HSTs produced by *Alternaria* species. Ch: chloroplast, ER: endoplasmic reticulum, GA: Golgi apparatus, Mt: mitochondrion, Nu: nucleus, Pd: plasmodesma, Pm: plasma membrane, Vc: vacuole.

**Fig. 2:**
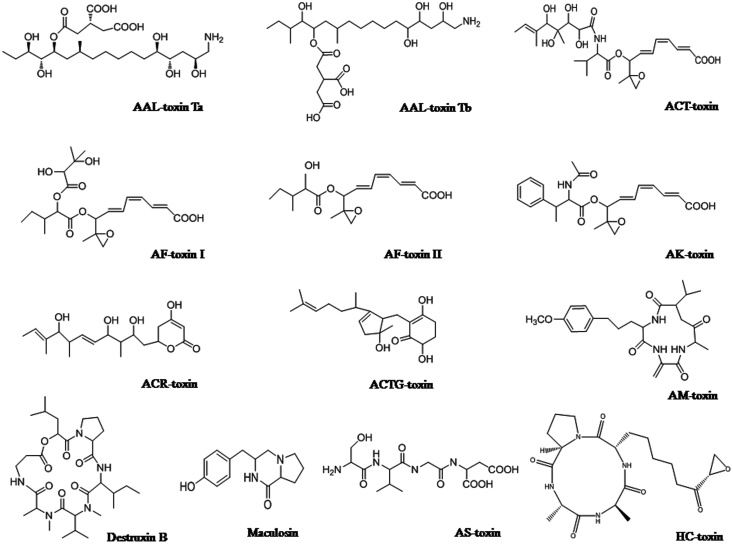
Chemical structures of host-specific toxins produced by various species of *Alternaria* (Modified of [4]).

There are many *Alternaria* toxins which have been described to possess cytotoxic, genotoxic, mutagenic, fetotoxic and/or teratogenic activity. In microbial and mammalian cell systems, these toxins cause mutagenic, oestrogenic and clastogenic effects by inhibiting the cell proliferation. In spite of the fact that *Alternaria* toxins can originate in almost entirely food and feed products and that they have the potency to exhibit harmful effects on human and animal health [35]. Despite the fact that, *Alternaria* toxins can originate in almost entirely food and feed products and that they have the potency to exhibit the harmful effects on human and animal health [[36], [37], [38]]. Right now, there are no precise international regulations or any national regulation in the world for any of the *Alternaria* toxins in food and feed, with the exception of Bavarian health and food safety authority. This authority decided the TeA content to limit (500 μg/kg) in sorghum/millet-based infant food [38,39]. Fig. 1 and Table 1 showed different HSTs related pathotypes, caused diseases, genes, chemical characteristics and their target site in the host plants, and the chemical structures of these different HSTs provided in Fig. 2.

**Table 1:** Showing host-specific toxins produced by *Alternaria* species, their host plants, chemical characteristics, responsible genes, and target site of these toxins (Source: Adopted from [4] with some addition).

*Alternaria* species (Pathotype)	Disease	Host range (susceptible cultivar)	Gene	Host-specific toxins	Chemical characteristics	Target site of toxin	Reference
*Alternaria alternata* f. sp. *lycopersici* (Tomato pathotype)	Alternaria stem canker of tomato	Tomato (Earlypack 7, First)	*ALT* genes	AAL-toxin Ta and Tb	Aminopentol esters	Aspartate carbamoyl transferase; sphinganine N-acltransferase	[26,27,4,5]
*Alternaria alternata* f. sp. *citri tangerine* (Tangerine pathotype)	Brown spot of tangerine	Targerines and Mandarins (Dancy, Emperor, Minneola)	*ACTT* genes	ACT-toxin I and II	Epoxy-decatrienoic esters	Membrane protein	[40,12]
*Alternaria alternata* f. sp. *fragariae* (Strawberry pathotype)	Black spot of strawberry	Strawberry (Morioka-16)	*AFT* genes	AF-toxin I, II and III	Epoxy-decatrienoic esters	Microsomal phospholipase A_2_	[41,42]
*Alternaria alternata* f. sp. *kikuchana* (Japanese pear pathotype)	Black spot of Japanese pear	Japanese pear (Nijisseiki)	*AKT* genes	AK-toxin I and II	Epoxy-decatrienoic esters	Sulfhydryl-containing molecules in membrane protein	[43,44,12]
*Alternaria alternata* f. sp. *citri jambhiri* (Rough lemon pathotype)	Leaf spot of rough lemon	Citurs rootstocks (Rough lemon)	*ACRT* genes	ACR(L)-toxin I	Terpenoid	Mitochondria	[45,46]
*Alternaria alternata* f. sp. *mali* (Apple pathotype)	Alternaria blotch of apple	Apple (Red Gold, Starking)	*AMT* genes	AM-toxin I, II and III	Cyclic peptide	Membrane protein; chloroplasts	[47,48]
*Alternaria alternata* f. sp. *longiceps* (Tobacco pathotype)	Brown spot of tobacco	Tobacco	*ATT* genes	AT-toxin	–	Mitochondria	[47,21]
*Alternaria alternata* (Spotted knapweed pathotype)	Black leaf blight of knapweed	Spotted knapweed	–	Maculosin toxin	Cyclic peptide	Ribulose-1,5-bisphosphate carboxylase	[49,50]
*Alternaria brssicae*	Gray leaf spot	*Brassica* species	*DtxS* genes	Destruxin A, B	–	Vacular H^+^-ATPase	[[51], [52], [53]]
*Alternaria alternata* (Sunflower pathotype)	Leaf spot of sunflower	Sunflower	–	AS-toxin I	Tetrapeptide		[54]
*Alternaria brassicicola* (*Brassica* pathotype)	Black leaf spot of *Brassica* spp.	*Brassica* species		AB-toxin	Protein		[55,56,^57^]
*Cochliobolus* (*Helminthosporium carbonum*) and *Alternaria jesenskae*	Leaf spot and ear rot disease in maize	Maize	*TOXE*, *TOXA*, *TOX2* genes	HC-toxin	Cyclic tetrapeptide	Mitochondrial membrane	[58,59]
*Alternaria brassicae*	–	–	–	ABR-toxin	–	–	[60]
*Alternaria panax*	–	–	–	AP-toxin	–	–	[19]

It is well-known that species in the *Alternaria* are versatile pathogens contaminating various crop plants, post-harvest fruits, refrigerated food products, as well as affecting different developmental stages of plants. The toxicity of *Alternaria* toxins has not hitherto been elucidated in detail for all substances and is still a matter of ongoing research. Therefore, this article provides knowledge about the toxicity of *Alternaria* toxins which will be helpful to the researchers and scientists who will work in this specific field. In this article, we review the most important *Alternaria* mycotoxins, their target sites in plant organelles, and the harmful effects of these toxins cause diseases on plants.

## 2 Host-specific (HSTs) toxins of *Alternaria* species

### 2.1 ACR-toxin

Leaf spot on rough lemon is caused by ACR-toxin. ACR-toxin I contain a dihydropyrone ring with C19 polyalcohol and toxicity of ACR is depended on pyrone ring with different polyalcohol side chain length and weaker toxicity [43,47,61,62]. Kohmoto et al. [63] have reported that ACR-toxin first targets the mitochondria and then other cell organelles. Using electron microscopy they were able to show that ACR-toxin enters the mitochondria through mitochondrial membrane pore [63]. ACR-toxin interrupts oxidative phosphorylation of mitochondria. The ACR-toxin has a similar mode of action to 2,4-dinitrophenol or carbonyl cyanide m-chlorophenyl that uncouple oxidative phosphorylation from ATP synthesis that disturbs membrane potential, which leads to NAD^+^ leakage from tricarboxylic cycle from susceptible variety lemon mitochondria [48]. A similar type of response to ACR-toxin as *Cochliobolus heterostrophus* race produced Texas cytoplasm male-sterile (T-cms) maize both will be affected the cell organelle mitochondria. Structurally, the similarities of ACR and T-toxin have polyols moieties and long-chain fatty acid polyketides [43,47,61]. Both toxins have host-specific toxins such as ACR for rough lemon, while T-toxins for *C. heterostrophus.* T-toxins have a protective effect on leaves, while ACR has a toxic effect on leaves, but ACR-toxin has no effect on T-cms maize [48]. A similar response to ACR-toxin and *C. heterostrophus* produced by T races Texas cytoplasm male-sterile (T-cms) maize both will be affected the cell organelle mitochondria by uncoupling of oxidative phosphorylation, stimulate NADH respiration, inhibition of respiration states, leakage of calcium, NAD^+^, and mitochondrial swelling.

*ACRT* gene responsible for the biosynthesis of ACR-toxins, *ACRTS1*, and *ACRTS2* genes encode a putative hydroxylase and PKS responsible for rough lemon pathogenesis confirmed through artificial technology of gene disruption and gene silencing methods [46]. *ACRTS1* and *ACRTS2* genes encode a putative hydroxylase and PKS, responsible for rough lemon pathogenesis. These genes have multiple copies on the same chromosome with 1.2–1.5 Mb size [64]. ACR-toxin has been isolated from the mitochondrial genome of rough lemon [48,65,66]. *ACRS* gene has responsible for ACR-toxin showed sensitivity to *Escherichia coli*, which was located in the group II intron of the mitochondrial tRNA-Ala due to alternative splicing of mitochondrial DNA sequence. This toxin is present in both toxin-sensitive and insensitive *ACRS* genes of mitochondria. *ACRS* genes have a shorter transcript for the mitochondrial sensitive plant. Due to the differential post-transcriptional processing of mitochondrial genes specificity of toxins is changed ACR-toxin specificity towards *A. alternata* on rough lemon pathotype and *Citrus jambhiri* [65,66].

ACR toxin causes pores forming on mitochondrial membrane proofed by several deletion experiments. ACRS transcripts having protein coding area responsible for the pore-forming transmembrane protein that is 171 bp for ACR-toxin, translated to 6683 kDa molecular weight product [65,66]. Three proteins with a molecular weight of 14, 21 and 28 kDa from mitochondria of rough lemon, during SDS-PAGE, proteins are not fully dissociated by immunoblotting maybe the dimer, trimer, and tetramer were identified by using ACRS antibodies [65,66].

### 2.2 ACR-toxin target site

Mitochondria are the primary target site for the action of ACR-toxin. When susceptible host treated with ACR toxins observed with swelled mitochondria characterized by partial destruction of the cristae, the disappearance of dense granules and mitochondrial membrane shows bulge formation [63]. ACR toxins also lead to uncoupling of the oxidative phosphorylation from the mitochondrial electron transport chains, loss of membrane potential, and most remarkably, the leakage of NAD^+^ from the Krebs cycle [48], resultant pores formation on the mitochondrial membranes [22]. Not remarkably, the gene in rough lemon (*ACRS*) which provides susceptibility to the ACR-toxin was observed to be in the mitochondrial genome. Transformation of *E. coli* with *ACRS* reduces them sensitive to ACR- toxin similarly. Although, the ACR toxin in plants are not determined any affects in the presence or absence of ACRS transcripts, but rather by a certain post-transcription alterations [65,66].

### 2.3 AAL-toxin

AAL-toxin was first isolated by Bottini and Gilchrist [40] from a tomato plant. They are chemically determined by propane 1,2,3-tricarboxylic acid (PTCA) which is the esterified form of 1-amino-11,15-dimethylheptadeca-2,4,5,13,14-pentol. There are five types of AAL toxins namely sphingosine (TA), phytosphingosine (TB), sphinganine (TC), tetra-acetyl-phytosphingosine N-lignoceroyl-d-erythro-sphingosine (TD), and L-sphinganine, each consisting of two isomers [4,67]. AAL toxins of TA and TB are structurally isoforms but differ at hydroxyl group at C4 and C5. TB is the N-acetylated form of TD, while TC is the N-acetylated form of TE [67]. TA and TB have considered as highly toxic activities as compared to TC and TD, therefore these two toxins are taken into consideration and referred to as AAL toxin. Among all AAL-toxins, TA-toxin has high toxic activity is more produced with the molecular weight of 522 KB [68].

**Fig. 3:**
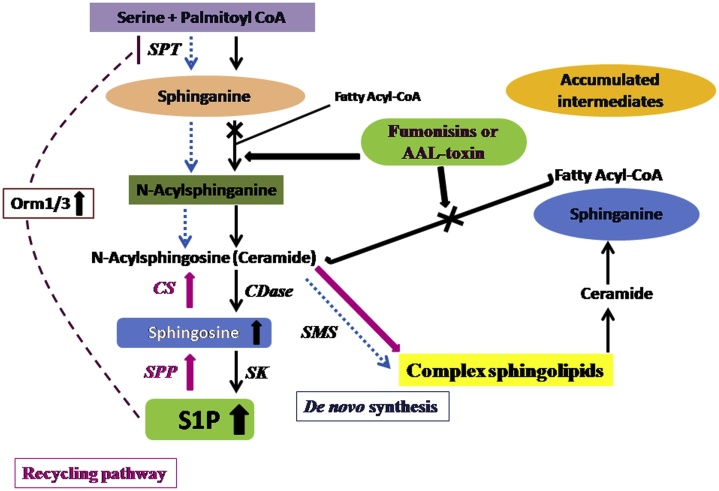
Schematic presentation of sphingolipids metabolism pathways and consequences of S1P lyase deficiency, and also presents the site of AAL-toxin/fumonisin inhibition. S1P lyase paucity leads to enhance of cellular S1P and sphingosine (to a smaller extent). Thus, *de novo* sphingolipid biosynthesis (blue arrows) is decreased may be up-regulation of Orm1/3 expression. At the same time, the recycling pathway (gray arrows) is elevated. SMS: sphingomyelin synthases, SMase: sphingomyelinases, CS: ceramide synthases, CDase: ceramidases, SPP: S1P phosphatase, SK: sphingosine kinases.

AAL-toxin is structurally analogue of sphinganine, and competent to prevent the enzyme sphingosine-N-acyltransferase (ceramide synthase, EC 2.3.1.24) in the endoplasmic reticulum, and therefore disrupt the breakdown of ceramide-containing lipids [69]. In the case of plants and animals, ceramide act as bioactive compounds which participate in numerous signaling processes as second messengers and thus regulate cell fate [70,71]. AAL-toxin treated tomato plant causes a hypersensitive response (HR) and involves ethylene, calcium, and MAP kinases (EC 2.7.11.24) [[72], [73], [74]]. Other signaling molecules taking part in the programmed cell death (PCD) cascade of reactions is nitric oxide (NO) and reactive oxygen species (ROS; especially H_2_O_2_; [75]) (Fig. 3). DNA laddering was observed on PCD and enhanced by Ca^2+^. Structure of AAL-toxins resemblance to the structurally related fumonisins induces an increase in the concentration of phytosphingonine and sphinganine in resistant tomato genotypes as well, but in this case, the effect is much less pronounced, indicate a higher *in vivo* inhibition of ceramide synthase in the sensitive plants [42]. The mechanism of AAL-toxin activity was that supplementing susceptible tomato leaves with ceramide that inhibit the AAL-toxin mediated PCD, the observation that ceramide balance is important for the onset of PCD [76]. However, AAL-toxin resistance tomato is conferred by the gene Alternaria stem canker resistance gene 1 (*Asc1*), that is a homologue of the yeast longevity assurance gene 1 (*Lag1*), which encoding ceramide synthase (CerS) isoenzyme [76]. Lag1 homologues determine the response to AAL-toxin and other SAMs also in other plant species, for example, the gene lag1 homologue 2 (*loh2*) in *Arabidopsis thaliana* [75].

AAL-toxin shows more host-specificity which causes necrosis and stem canker on tomato plant of the *asc/asc* genotype [77]. At the point when imposed by AAL-toxin, vulnerable tomato tissues aggregate phytosphingonine and sphinganine [42], although multifaceted sphingolipids compounds are depleted simultaneously [78]. Effect of AAL-toxin stops ceramide synthesis by inhibiting key enzyme sphinganine N-acyltransferase (acyl-CoA-dependent ceramide synthase) [79,80]. Pyrimidine metabolism is disrupted by AAL-toxin effects on enzyme activity aspartate carbamoyltransferase (ACTase). Two amines ethanolamine (EA), phosphoethanolamine (PEA) are accumulated after treatment with AAL-toxins with tomato susceptible plant. In the biosynthetic pathways, EA and PEA are the primary and secondary metabolite intermediates.

### 2.4 AAL-toxin target site

AAL-toxin affects organelles like mitochondria and endoplasmic reticulum; moreover, their accurate target site is still unknown. *ALT1* gene is responsible for AAL-toxin biosynthesis that encodes a polyketide synthase gene (*PKS*) and other related toxins such as ACT-toxin, ACTG-toxin, ACR-toxin, AM-toxin, AS-toxin, tentoxin, AF-toxin, AM-toxin, AK-toxin, brefeldin, maculosin, and destruxin B which accumulation occur in leaves after treatment with AAL-toxin. It has been reported that homozygous resistant and homozygous susceptible genotype that is cell-free ACTase of host-specific and non-host-specific resource communicate inconsistency of AAL-toxin sensitivity. Usually, AAL-toxins affect the mitochondria but their accurate target site is still doubtful. The potential biosynthetic pathway of AAL-toxin is under exploration as the primary and secondary intermediary metabolites of biosynthetic pathways of EA and PEA [4].

The induced PCD was occupied by DNA laddering, chromatin condensation, cell shrinkage, TUNEL-positive cells, and the development of apoptotic-like bodies [26]. Due to AAL-toxin mediated PCD includes cell cycle disruption and ceramide signaling [17]. AAL toxins induce physiological and development effects of necrotic lesions on fruits and leaves, inhibition of *in vitro* development of calli, pollen, roots, and shoots, and also decreases the viability of protoplasts and suspension cells [81]. AAL-toxin disrupting sphingolipid metabolism which promotes programmed cell death in tomato leaves promoted by ethylene and jasmonic acid and *Asc* gene is responsible for sphingolipid biosynthesis [82].

According to Zhang et al. [82], jasmonic acid and ethylene-dependent pathways triggered by programmed cell death by AAL-toxin *via* sphingolipid metabolism disruption in the tomato plant, whereas according to Akamatsu et al. [83] AAL-toxin lacking REMI mutants are non-pathogenic in the tomato delicate plants. Insertion study of toxin-deficient isolates proficient to the identification of the *ALT1* gene encoding a group I polyketide synthase that is intricate in AAL- toxin synthesis [83,84]. *ALT1* function was affirmed by hereditary complementation of *Fusarium verticillioides* and *Gibberella moniliforme* in which FUM1 mutant damaged in fumonisin production [85]. Despite the fact that AAL- toxin and fumonisins share fundamental features and therefore show comparable physiological impacts, Fum1-inferred fumonisin delivered by *F. verticillioides* was appeared to be dispensable for maize disease infection [86]. Remarkably, *Gibberella moniliforme*, the causal specialist of maize seedling blight produces mycotoxin like fumonisin B1 belongs to the group of polyketide produces by *Fusarium* species. Fumonisin B1 toxin triggered programmed cell death with disruption of the vacuolar membrane by lesion formation [87]. Fumonisin-unaffected maize plants are not resistant to infection of disease albeit systemic colonization of seedlings by *G. moniliforme* is decreased [88]. As ceramides are by all account not the only crucial constituents of cellular membranes yet, in addition, intracellular signaling molecules, signal transduction, and regulatory processes may likewise be influenced.

AAL-toxin is effective as herbicide from a compound of *A. alternata* (Fr.) at very small concentrations against a wide range of leaf plants (e.g. jimsonweed, prickly sida, and black nightshade). However, in the case of monocotyledonous crops, for example, maize, wheat, and some varieties of tomato are tolerant to AAL-toxin [89]. The effect of AAL-toxin on duckweed (*Lemna pausicostata* L.) illustrated cellular electrolyte leakage and loss of chlorophyll content at the concentration of 20–40 nM after 72 h treatment [90]. Similar types of results were found on the susceptible variety of tomato. The first symptom causes at the ultra-structural plane with the distraction of the plasma membrane, fast cellular leakage of electrolytes and collapse at the cellular level. Toxin effects are become visible and associated with plasma membrane dysfunction, and these effects are not dependent on the light. Fumonisins and sphingolipid bases like phytosphingosine are less potent but cause similar effects (about 10-fold of fumonisins bases, and about 100-fold of sphingolipid bases) [90]. AAL-toxin and fumonisin B induced interruption of sphingolipid metabolism cause phytotoxic injury and cell death on the susceptible varieties tomatoes and duckweeds.

### 2.5 AM-toxin

AM-toxin is also a host-specific phytotoxin causing leaf spot on Apple is known as *Alternaria mali*. There are three host-specific AM-toxins; AM-toxin I, AM-toxin II, and AM-toxin III. AM-toxin I having cyclic tetradepsipeptide containing an _L_-2-hydroxy-3-methylbutanoic acid (_L_-Hmb) residue, along with two unusual amino acids [α, β-dehydroalanine (ΔAla) and _L_-2-amino-5-(*p*-methoxyphenyl)pentanoic acid (_L_-Amp)] [91,92]. AM-toxin I is comprised of four structural chemical compounds, such as α-amino acrylic acid, _L_-alanine, _L_-α-hydroxy-isovaleric acid, and _L_-α-amino-δ-(*p*-methoxyphenyl)-valeric acid. AM-toxin II and III, _L_-Amp of AM-toxin I is replaced by _L_-2-amino-5-phenylpentanoic acid (_L_-App) and _L_-2-amino-5-(p-hydroxyphenyl) pentanoic acid (_L_-Ahp), respectively. The target site of AM-toxins on apple susceptible cell on chloroplast by reduces chlorophyll content by affecting grana lamella and chloroplast disorganization and inhibiting photosynthesis and another organelle is the plasma membrane [93,94]. Cyclic peptides of AM-toxin are produced by non-ribosomal pathways by vast multifunctional enzymes called cyclic peptide synthetases (CPSs) pathway having a conserved area [95]. *AMT1* gene responsible for AM toxin synthesis that encodes 479 kDa nonribosomal peptide synthetase (NRPS) consists of four catalytic domains responsible for activation of each residue in AM-toxin. Genes responsible for AM toxin synthesis are *AMT2*, *AMT3*, and *AMT4* [96]. Strain IFO8984 chromosome contains 1.3 Mb with multiple sets of AMT clusters [97], more than 10 putative clusters present on the same chromosome, AMT region is enriched in transposons fossils resembling the strawberry pathotype [97].

### 2.6 AM-toxin target site

The hitherto described *Alternaria* EDA-derivative HSTs, the depsipeptide AM-toxin has two target locations: the chloroplasts and the plasma membrane. The properties on the plasma layer are reminiscent of those influenced by the EDA HSTs. Moreover, AM-toxins are categorized by their extremely detrimental subtype; the AM-toxin has a place with the classification of a considerably-destructive subtype [98]. At the lower content of AM-toxins causes the polysaccharide exudates and membrane fragments. AM-toxins triggered decrease polysaccharide inclusion due to decreased activity of the Golgi complex, despite the similar mechanism of modifying the membrane. The AM-toxin induces disorganization of the chloroplast and ultra-structural changes due to the interference of the grana lamellae, which leads to the emergence of membrane fragments and vesicles in the stroma. AM toxin causes chloroplast disorder is well-matched with a decrease in the chlorophyll substance and restraint of photosynthetic CO_2_ absorption action on toxin treated susceptible leaves [99].

### 2.7 AT-toxin

AT-toxin is produced by tobacco pathotype, the causal fungus of brown spot disease in the tobacco plants. AT-toxin is host-specific and the target to mitochondria on susceptible cultivar Burley 21, causing mitochondrial bulge formation, and mitochondrial membrane attenuation [98,100]. AT-toxins influence the physiology of plant causing necrotic, chlorotic halo zone formation on leaves of the susceptible plant. Experimental work was done on the tobacco leaves that affect the plant physiology by increasing stress metabolites such as hydrogen peroxide (H_2_O_2_), proline content, and ROS level. One of the applications of AT-toxin is that it suppresses the programmed cell death when applied to leaves having some inhibiting compounds like caspase specific peptide inhibitors, serine protease, nuclear lamina, poly (ADP ribose) polymerase, and topoisomerase I [101].

### 2.8 AT-toxin target site

The HST of *Alternaria longipes* effects the mitochondrial ultrastructural modification has a similar effect as ACR toxins [98,100,101]. AT-toxin application leads to increases H_2_O_2_ accumulation as well as an increase in the quantities of stress-related compounds like malondialdehyde and free proline, and also protease activity was proved by molecular level [102]. In addition, the appearance of lesions and stress markers generation can be inhibited by pre-infiltration of the susceptible tobacco tissues with caspase-specific peptide inhibitors. These observations show that ROS-homeostasis and caspase-like proteases have a crucial role in the PCD process mediated by the AT-toxin [102].

### 2.9 AF-toxin

AF-toxin cause’s black spot on strawberry is host-specific toxin produced by *A. alternata* and encoded by AFT gene. AF-toxin showed highly susceptible to strawberry roots than leaves of the susceptible plant [103]. Three molecular species of AF-toxins has been reported as AF-toxin I, II, and III. AF-toxin I also showed more toxicity towards pear and strawberry [26,104]. AF toxin III is highly toxic towards strawberry and less toxic to pear while AF-toxin II is toxic to pears [105]. Derivatives of AF-toxin I and III are 2,3-dihydroxy-isovaleric acid and 2-hydroxy isovaleric acid both are valine derivatives while AF-toxin II has 2-hydroxy valeric acid which is a derivative of isoleucine [26]. Hatta et al. [106] determined the structure of AF-toxin which depends on the basis of 1.0 Mb chromosomal strain of NAF8 and establish 2–7 copies of 20 AFT areas. AF-toxin biosynthesis gene *AFT1*, *AFT2* isolated from the NAF8 strain, there are 11 copies of AFT genes and 5 transposons like sequence, TLS1-TLS5.

### 2.10 AF-toxin target site

AF-toxin primary target organelle is plasma membrane causing membrane invaginations, vesiculation, fragmentation, and depolarization that leads to decreases in membrane potential gradients, and AFT gene is responsible for AF-toxin biosynthesis. Conditionally dispensable chromosome (CDCs) is encoding AFT genes. A few minutes of toxin treatment with AF-toxin increases of K^+^ efflux on the plasma membrane of susceptible cell compartments [41,98,105,107]. This toxins effect polarization of the plasma membrane is a respiration-reliant component of membrane potential. Only plasma membrane of a cell is affected by AF-toxin but no further intracellular organelles affected. It indirectly affects the plasma membrane H^+^-ATPase of the susceptible plant [20,22]. After 1–3 h toxins treatment, the fusion of Golgi vesicles causes the damage of membrane [98].

### 2.11 AK-toxin

Toxicity of AK-toxin formed through Japanese pear pathotype of *A. alternata* causes the black spot on pear [8]. The Japanese pear pathotype secretes two associated molecular classes, AK-toxins I and II, amid toxin I being more abundant and biologically active species [47]. Both toxins reveal toxicity only on susceptible pear cultivars [108]. AK-toxins are the esters of 9,10-epoxy-8-hydroxy-9-methyl-decatrienoic acid (EDA) which are derivative of phenylalanine and hydroxyl decartienoic acid. Based on restriction enzyme-mediated integration (REMI), a transformation was benefited to isolate AK-toxin-minus mutants (Liu et al., 2014). Genes responsible for AK toxins biosynthesis (*AKT1*, *AKT2*, *AKT3*, *AKT4*, *AKTR*, and *AKTS1*) are essential for structural and functional analysis by cloning strategy [109].

*AKTR* transforms a putative transcription regulator having a zinc binuclear cluster DNA-binding domain distinctive of the fungal Zn(II)2Cys6 family of proteins [4,110]. The transcriptional factors with this domain control various primary, secondary metabolism, and drug resistance in fungi [58]. Imazaki et al. [111] described that the enzyme responsible for AK-toxin biosynthesis consists of signal type 1 (PS1)-like tripeptides, SKI, SKL, and PKL at the C-terminal ends that is responsible for peroxisomal localization. Mutation of *AaPEX6*, which encodes a peroxin protein responsible for peroxisomes biogenesis, in the Japanese pear pathotype resulted in lack of functional peroxisomes and fully loss of AK-toxin production and pathogenicity [111]. AK-toxin effects the plant physiology by causing abscission of immature fruit and discoloration on mature fruits leads to extensive fruit losses, affect young leaves, brown spot develop on infected leaves. AK-toxin I and AK-toxin II cause leave necrosis and rapid efflux of K^+^ from the plasma membrane of the susceptible host [17,112].

### 2.12 Maculosin toxin

Maculosin [the diketopiperazine, cyclo (_L_-Pro-_L_-Tyr)] is a host-specific phytotoxin produced by *A. alternata* on spotted knapweed (*Centaurea maculosa*) [52]. Its distinctive selectivity, apparent safety, and simple structure make maculosin a perfect chemical principal for developing an innocuous, safe and environmentally approachable anti-knapweed herbicide [113]. After three-five days of treatment with *A. alternata*, causes chlorotic spots emerging into black necrotic lesions on the leaves of knapweed with the production of frequent, non-toxic diketopiperazines, maculosin [cyclo (_L_-Pro-_L_-Tyr)] [52,98,114]. Several cultivars of spotted knapweed significantly differ in their response to maculosin which recommend the amendment of the toxin possibly occurring to yield bio-inactive metabolites [115].

In the examining of diketopiperazines, it is clear that the mycotoxins activity needed certain functional groups [52]. Maculosin is the most vigorous mycotoxin containing a phenolic moiety [115]. Studies on diketopiperazines including proline designated that _L,L_ compounds recognize an extended confirmation while the _L,D_ diastereomers would have a more folded conformation [53]. Stierle et al. [52] have isolated several diketopiperazines from liquid cultures of *A. alternata,* the causal agent of black leaf blight of spotted knapweed, *Centaurea maculosa* Lam. The compounds were first applied to knapweed leaves and hypocotyls that induced lesions at 10^−3^, l0^-4^, and 10^-5^ M. The compounds cyclo (-_L_-Pro-_L_-Phe-) and cyclo (-_L_-Pro-_D_-Phe-), differed in phytotoxicity: the _L,L_ diastereomer influenced necrotic lesions on knapweed leaves at 10^−3^ M, but the _L,D_ isomer was not vigorous, even at 10^−3^ M. Maculosin (cyclo-Pro-Tyr) is an ideal prototype for creating a safe and environmental friendly anti knapweed herbicide. To evaluate this possibility, Bobylev et al. [113] synthesized and tested a series of 18 maculosin analogs by the use of spray or brush application in the greenhouse conditions on the entire knapweed plants. Interestingly, there were many of the maculosin analogs which have substantial potential as natural herbicides against spotted knapweed. One of the simplest analog (cyclo-Pro-Phe) eradicated two-thirds of the spotted knapweed at the concentration of 6 × 10^-2^ mol/L within 15 days.

### 2.13 Destruxin-B

Destruxin was first isolated from *Metarrhizium anisopliae* [116]. Destruxin-B is a host-specific toxin produced by *Alternaria brassicae* which causes gray leaf spot on *brassica* plants. Destruxin-B isolated by spore germination fluids methods. Destruxins are hexadepsipeptides hydroxyl acid composed of five amino acid residues with a molecular weight of 593 kDa. Destruxin B is the vital phytotoxin formed by the pathogen in liquid media, and the three additional phytotoxins, homodestruxin B, destruxin B2, and desmethyldestruxin are formed in much smaller amounts [51,[117], [118], [119]]. On the basis of different hydroxyl acid, N-methylation, R-group of amino acid residue are S, R with hydroxyl acids. Destruxin A–E with same amino acid sequence but differ R group of the hydroxyl acid residue. Destruxins, where the proline (Pro) residue (n=3) is substituted with a pipecolic acid (Pip) residue (n=4) was selected by the identical letters with the subscript 1, i.e. A1–E1, although destruxins amid a valine (Val) residue (R0=CHMe2) as a substitute of the isoleucine (Ile) residue (R0=CHMeCH2Me) were selected with subscript 2, i.e. A2–E2 [120].

Dextruxin-toxin causes necrosis and chlorosis on the non-host-specific plant. Electron microscopy of healthy, chlorotic and necrotic portions of *Brassica campestris* leaves naturally infected with *A. brassicae* revealed considerable differences at ultrastructure level. The necrotic lesions showed plasmolysis with total disruption of cell organelles. The chlorotic lesions had normal plasma membrane but swollen mitochondria with a reduced number of cristae and vesiculation of the envelope. Chloroplasts showed degeneration of granal fretwork with an increase in the number of plastoglobuli. Chlorotic lesions due to foliar application of destruxin-B induced identical changes in leaves.

### 2.14 ABR-toxin

ABR-toxin is a host-specific toxin that is caused by *A. brassicae* with a water soaked disease symptoms followed by chlorosis in *Brassica* leaves [121]. The toxin in spore germination fluid (SGFs) collected after inoculation of *A. brassicae* on host leaves was retained by ultrafiltration with a 10 kDa cut off membrane and the activity was abolished by temperature and proteinase K treatments, showing that dissimilar to different toxins responded to be formed by *A. brassicae* [118,122]. For purification of the toxin, ammonium sulfate fractionation and IEC were effective stages for eliminating the yellow pigmentation and several contaminating proteins deprived of any toxicity. ABR-toxin moderately purified by HIC demonstrated a consistent band of 27.5 kDa molecular weight recognized through SDS-PAGE electrophoresis and this band was related to the toxicity on host plants. Ultimately, GFC and HPLC affirmed that the 27.5 kDa protein was related to the toxicity. ABR-toxin at concentrations of 0.5–1.0 μg/ml prompted water-soaked symptoms subsequently chlorosis on *Brassica* leaves, although non-host leaves were not affected uniform at 50 μg/ml indicating host-specific toxicity. Consequently, ABR-toxin does not only induce the preliminary colonization in host plants, but also appears to be involved in disease enlargement [123].

Till date, the complete amino acid sequence of ABR-toxin was not determined so far; therefore the N-terminal 21 amino acid residues show vast similarity with trypsin precursor of *F. oxysporum* in the sequence database search. Thus, ABR-toxin encoded gene cloning is currently being endeavored [21,110,118,123,124].

### 2.15 AB-toxin

AB-toxin is host-specific toxin responsible for black spot disease on *Brassica* plants known as *Alternaria brassicicola* [4,124]. The molecular weight of AB toxin is estimated to be 35 kDa by SDS-polyacrlamide gel electrophoresis and most of the AB-toxin is produced by SGFs [21]. Toxin purified by ion exchange chromatography and gel filtration HPLC. Oka et al. [125] described that production of AB-toxin by propagating spores of *A. brassicicola* is prompted by recognition of host-derivative oligosaccharide of 1.3 kDa. The similar oligosaccharide resulting from host leaves may be convoluted in the production of ABR-toxin by germinating spores of *A. brassicae*. AB-toxin activity was heat labile and was also lost after treatment with proteinase K [124].

In *Brassica* plants, both the pathogens (*A. brassicae* and *A. brassicicola*) have pathogenic with the similar type of host range, and ABR-toxin and AB-toxin have equivalent host-specific activity, but vary in molecular weight. The mode of action of ABR-toxin and AB-toxin on plants is not known yet. Therefore, in future research, it will be significant to study the mode of action of AB-toxin and ABR-toxin on host plants.

### 2.16 AS-toxin

Two phytotoxins are isolated from *A. alternata* culture filtrates which pathogenic to sunflower. One was recognized by chemical and physicochemical strategies as the tetrapeptide Ser-Val-Gly-Glu [4,55,56]. This peptide name is AS-I toxin. This toxin causes chlorosis or necrosis on leaves and inhibits seed germination. Currently, there is very less information available for this toxin. So, in future research, there will be scope for researchers to study this particular toxin and their mode of action.

### 2.17 AP-toxin

AP-toxin is also host-specific toxin which is produced by *Alternaria panax*, the causal agent of *Alternaria* blight of *American ginseng* [21]. The molecular weight of AP-toxin is also 35 kDa, which is similar to the molecular weight of AB-toxin. AP-toxin does not persuade any kind of symptoms on the hosts of *A. brassicicola*. AP-toxin produced by *A. panax* is dissimilar from the AB-toxin produced by *A. brassicicola* [21].

## 3 Mode of action of host-specific toxins (HSTs)

Albeit, the site of action of different *Alternaria* toxins differs, but the ultimate end of the toxins is to trigger the host cell death.AK-, AF-, and ACT-toxins perform to have an initial effect on the plasma membranes of susceptible cells and cause permeabilization [20,126,127]. An expansion in electrolyte damage from tissues and invagination of plasma membranes is a typical trademark of toxin action. These progressions are apparent within 1 h after exposure to the toxin. AM-toxin exposed the harmful effects not only the plasma membrane but also on chloroplasts, whereas ACT-and AT-toxin were found to affect mitochondria [20]. Besides, AK- and AF-toxins stimulate a depolarization of membrane electropotential in 5–10 min [128,129].

ACR-toxin initiates swelling and other morphological alterations of mitochondria and expands NADH oxidation, which is monitored by plasma membrane ailments prompting electrolyte leakage/discharge and necrosis [48]. For most of these toxins, still, their action mechanism is known only for barely. On the other hand, Park et al. [130] predicted the ultrastructural destinations for electrolyte leakage in susceptible cells treated with AK- and AF-toxins by analytical electron microscopy and ion-precipitation approach. Precipitation of magnesium and sodium ions discharged from plasma membranes performs in cell walls adjacent plasmodesmata within 5 min, and soon after, the plasma membranes move toward becoming invaginated at both ends of the plasmodesmata, showing that the destinations of action for these toxins might be situated on the plasma membrane close to plasmodesmata.

The association between AK-toxin action and stimulation of fungal dissemination/penetration and colonization has been incompletely characterized by considering at balancing impacts of different treatments of toxin and fungal infection. SH-alkylating reagents had a stamped defensive impact on AK-toxin influenced electrolyte damage and veinal necrosis only when the leaves were treated hitherto exposure to the toxin. AK-toxin activated electrolyte damage also was suppressed by previous and concurrent treatments with nitrogen (N_2_) gas. Pear leaves pretreated with inhibitors of mRNA synthesis or protein syntheses were shielded from toxin-induced decay/necrosis but not from electrolyte damage. The defensive impact of the inhibitors on necrosis was distinguishable even when they were controlled 5 h after toxin exposure. Treatment with copper- and iron-chelating mediators had the same defensive impact as that of mRNA synthesis and protein synthesis inhibitors, however, the effect was distinguishable even 10 h after toxin introduction. The chemically treated leaves were immunized with the pathogen and fungal actions on the leaves were examined. SH-reagents vitally decreased the fungal infection and necrotic lesions, however inhibitors of mRNA synthesis and protein synthesis and copper- and iron-chelating operators did not ensure against fungal infection.

**Fig. 4:**
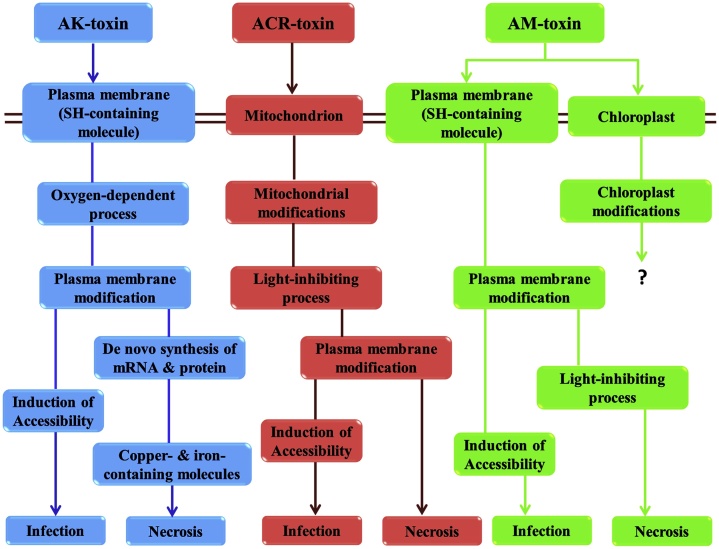
Summary through the diagrammatic presentation of the mode of actions of AK-, ACR- and AM-toxins in susceptible plants.

Based on data information, a hypothetical scheme for the mechanism of AK-poison action in susceptible pear is revealed in Fig. 4. In this way, AK-toxin has pleiotropic effects on susceptible cells after a specific response at the site on the plasma membrane, and dysfunction of plasma membrane is a vital event for induction of accessibility. Moreover, a deprived knowledge of the mechanism of action of various host-specific toxins, the principle of host-specificity is understood for some of them. For destruxin B, a host-specific phytotoxin formed by *Alternaria brassicae* [[131], [132], [133]] the mechanism of action for host-specificity was recently discovered. The specific phytotoxicity gives off an impression of being because of quick and effective detoxification, by successive hydroxylation and glycosylation responses, in tissues of resistant species. These responses were additionally found to occur in susceptible species, however, at a deliberate rate, providing clarification to specific toxicity [134]. Likewise, toxin detoxification has been revealed to regulate resistance against the host-specific toxin-producing fungus *Cochliobolus carbonum* [135].

For ACR-toxin, specificity appears to be controlled by differential post-transcriptional processing of a mitochondrial gene. This gene is available in the mitochondrial DNA of toxin-sensitive along with resistance species, however, the transcript of the gene is shorter in resistant than in sensitive mitochondria. Finally, an oligomeric protein is delivered in toxin-sensitive mitochondria, while the transcript is not transformed in resistant mitochondria. Despite the fact that the gene is apparent to encode a mitochondrial membrane protein, no function has been assigned so far [65,66].

## 4 Toxicity of *Alternaria* HSTs on animals

Toxicological data are inadequate to the above-mentioned major *Alternaria* toxins, and even these data are insufficient with virtuous bioavailability and long-term clinical studies [136]. Even though, there is little knowledge hitherto about their chemical, physical properties, and toxicological mechanisms, bioavailability, and stability in the digestive tract. These toxins expose detrimental effects in animals, comprising fetotoxicity, cytotoxicity, and teratogenicity [137,138]. These effects have been related to a range of pathologies from hematological diseases to esophageal cancer. Moreover, these effects may be mutagenic, estrogenic and clastogenic, in microbial and mammalian cell organisms and tumorigenic in rats [29,36,37,139,140].

The benzopyrone (AOH, AME, ALT, and AS toxins) is the most studied group amid all the *Alternaria* toxins. Although, this group toxicity is not completely understood and differs from one cell organelle to another, but AOH and AME toxicity have been recognized in several *in vitro* and *in vivo* systems [141,142]. AOH has estrogenic potential and inhibits cell proliferation [143], also AOH induces phenotypic changes in mice macrophages, which could not be directly related to early AOH-induced ROS production, cell cycle arrest or autophagy as seen as a consequence of AOH-induced double-stranded DNA breaks [144]. AME and AOH were not very extremely toxic, but they do exert genotoxic, mutagenic, carcinogenic, and cytotoxic in mammalian and microbial cell culture. Furthermore, AOH and AME were competent to induce gene mutations and DNA strand disruption in cultured human and animal cells [145,146]. These toxins also inhibited the activity of human topoisomerases by disrupting the stability of topoisomerase II-DNA-intermediates and DNA integrity by variation of the redox balance in human colon carcinoma cells [147].

The perylenquinone derivatives [ATX I, ATX II, ATX III, Alterperylenol (ALTCH; synonym Alteichin), and stemphyltoxins (STE)] are measured to be very dangerous because of their mutagenic properties [148]. Because of the absence of existing position complexes, in specific for ATXs, analytical studies persist less common [149]. Recently, ATXs have been reported to be extremely active mutagens and more severely toxic to mice and cause DNA strand disruption. In recent times, more genotoxic effectiveness of ATX II in both mammalian and human cells was confirmed, and it was pronounced as the furthermost effective substance within the ATX group, accomplished of different mechanisms of action [148]. Furthermore, data regarding the fundamental modes of action are still inadequate [147].

*Alternaria alternata* f. sp. *lycopersici* toxins (AALs) expose commonly phytotoxic effects but have been shown to interrupt the sphingolipid metabolism in an equivalent way to fumonisins, which have been associated with esophageal cancer and other animal diseases [42,150]. Altenusin (ALT) is a biphenyl derivative having antioxidative properties and capability to prevent several enzymes, for instance, sphingomyelinase, HIV-1 integrase, myosin light chain kinase, acetylcholinesterase, and cFMS kinase, trypanothione reductase, and pp60c-SRc kinase in the small micromole concentration range, and it can support as a chemotherapeutic agent to treat leishmaniasis and trypanosomiasis [151,152]. The biphenyl elementary skeleton of ALT comprising a salicylic moiety and a catechol moiety may possibly be the important part because of its interesting azole-synergistic activity.

The cyclic tetrapeptide tentoxin (TEN) is one of the most important *Alternaria* toxins formed, accompanied by dihydrotentoxin (DH-TEN) and isotentoxin (iso-TEN). Their structures fluctuate at the unsaturated bond of the N-methyldehydrophenylalanine moiety, which is hydrogenated into a single bond in DH-TEN and E configured in iso-TEN. Tentoxin (TEN) and their derivatives compound are considered to be phytotoxins, but TEN existences are the most effective, preventing photophosphorylation and persuading chlorosis. Nevertheless, no toxicological data are existing for mammals, and the data on the occurrence of this toxin in food and feed are limited also [149]. *In vitro* studies have revealed that AOH and AME were eagerly changed to glucuronides upon development with hepatic and intestinal microsomes from humans, rats, or pigs in the occurrence of UDP-glucuronosyltransferases (UGT).

## 5 Role of reactive oxygen species (ROS) during plant-pathogen interaction

In plant, under normal physiological process, ROS are produced through the process of molecular oxygen assimilation and under stress condition, rigorous ROS production is done. ROS molecules have a chief role in plant physiological activity like plant growth development and oxidative burst have a direct effect on pathogens or defensive activity [153].

ROS could directly kill the pathogen, in the form more reactive species like hydroxyl radicals, MDA and H_2_O_2_ in cell apoplast produced in response to pathogens. Various enzymes take part in apoplastic ROS production such as plasma membrane NADPH oxidase and cell wall peroxidase are main biochemical sources [154]. ROS produced under biotic stress condition have a very deleterious effect on plant cell components inside them such as protein, lipids, DNA which ultimately leads to plant cell death. While pathogen recognition occurs at apoplast, leads to primary oxidative burst, simultaneously ROS production also takes place in other cell organelles like chloroplast and mitochondria. Through activation of SIPK/Ntf4/WIPK cascade by pathogens, chloroplast ROS production unregulated, which plays a significant role in signaling or HR-mediated cell death in plants [155]. In plant cell, chloroplast oxidative burst, NADPH oxidase burst and mitochondrial ROS generation promote cellular apoptosis process.

For normal cell functioning, a balance between ROS production and elimination is needed. Plants have the ability to detoxify these ROS, by producing differential antioxidative enzymes mediated by SA [[156], [157], [158]]. Developed effective mechanisms have two components (i) enzymatic such as superoxide dismutase (SOD), guaiacol peroxidase (GPX), Catalase (CAT), glutathione reductase (GR), ascorbate peroxidase (APX), monodehydroascorbate reductase (MDHAR), and dehydroascorbate reductase (DHAR); (ii) non-enzymatic antioxidants like reduced glutathione (GSH), ascorbic acid (AA), α-tocopherol, carotenoids, flavonoids, and the osmolyte proline for scavenging pathogen attack [133,[159], [160], [161], [162]].

In regular metabolism, plant cells produce detrimental ROS molecules such as H_2_O_2_, singlet oxygen (^1^O_2_), the superoxide anion (O_2_**˙**ˉ) and the hydroxyl radical (**˙**OH) as by-products. ROS can be performed as upstream and/or downstream of many signaling cascades and the degree of accumulation determinates their function into the cell because they might take action as essential signal transduction molecules (at low levels) or as toxic molecules (at high levels) with strong oxidant power [108,163]. Fungal pathogens are exposed to the oxidative stress generated by ROS and evolved numerous traditions to scavenge ROS using small molecules (ascorbic acid, glutathione, flavonoids, alkaloids, and carotenoids) that will be oxidized by ROS, in addition to detoxifying enzymes (catalase, peroxidase, superoxide dismutase, and peroxiredoxins). To evaluate the importance of ROS for plant defenses is to impede with the mechanisms deployed by pathogens that defend them against ROS, but it can also be sensed by fungal pathogens and function as developmental signals for the differentiation of infection structures [[164], [165], [166], [167]].

**Fig. 5:**
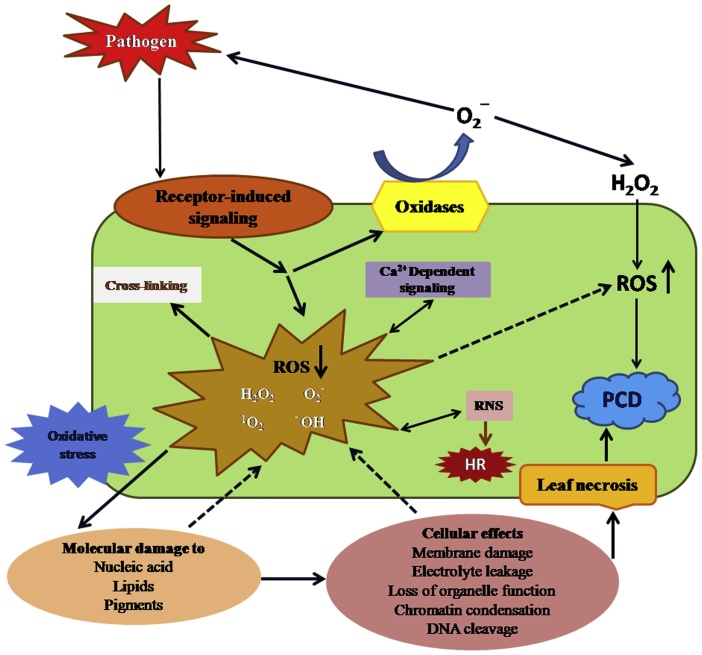
Role of ROS when pathogen attack situation (↑ represents up-regulation of ROS production, whereas represents down-regulation of ROS scavenging mechanisms). ROS play multifaceted action. The most important ROS-induced mechanisms during plant-pathogen interaction are peroxidase- and ROS-induced cross-linking of cell wall components which play an important role in the defense mechanisms against the pathogens. Furthermore, a defence-induced PCD, known as HR is stimulated and organized by the intricate crosstalk between ROS and RNS. In conclusion, ROS can be transform several other multiple signaling pathways and cell to cell reactions persuaded by various biotic and abiotic stimuli, by the oxidation-dependent regulation of transcription factors and by the co-induction and co-regulation of the secondary messenger Ca^2+^. PCD: programmed cell death, ROS: reactive oxygen species, RNS: reactive nitrogen species, H_2_O_2_: hydrogen peroxide, O_2_^‾^: superoxide radicals, HR: hypersensitive response. *Note: For more information about the role ROS see review, Apel and Hirt [180].

The ROS accumulation during avirulent pathogenesis precedes the HR and cell death that frequently come with successful pathogen recognition leading to incompatible interaction [168]. During plant-pathogen interaction, ROS accumulation has been anticipated as initial procedures influenced the growth of the pathogen. ROS have been proposed several significant roles such as antimicrobial molecules, plant cell wall cross-linkers blocks pathogen entry that acts as a local and systemic secondary messengers to activate additional immune responses, like stomatal closure or gene expression [[169], [170], [171], [172], [173], [174], [175]]. Besides of ROS accumulation, Ca^+2^ also play a vital role as a secondary messenger during several biotic and abiotic stress conditions. While, ROS and Ca^+2^ are co-produced and co-regulate each other, the analysis of the regulation of these pathways is complex [[176], [177], [178], [179]]. It is critical to stain that the ROS accumulation into the cell as detrimental, defensive/protective or signaling aspects depends on the sophisticated stability between ROS generator/scavenger systems at the appropriate position (Fig. 5).

## 6 Conclusion

*Alternaria* toxins are well recognized as a vital determinant of pathogenicity in plants. Host-specific toxins of various *Alternaria* species contribute a crucial role in pathogenesis and possibly will be applied as a discriminating agent in *in vitro* selection at the cellular stage for disease resistance. The function of a toxin as a disease establishment is confirmed by the degree of the toxin in infected plants and the competency of the toxin alone to elicit at least part of the symptoms of the disease. In this article, we have been studied various host-specific mycotoxins produced by *Alternaria*, chemical structure of HSTs their chemical property, host plant, HSTs biosynthetic genes, target sites organelle in plant and mode of action of HSTs. With widespread occurrence of *Alternaria* mycotoxins on crop plants and their consumptions through various animals and human being due to its highly toxic effects on plants and economic loss, more toxicological studies are needed. This review article will provide an idea to understand the process of disease development during exposure of pathogen or their toxins.

## Future prospects

*Alternaria alternata* is basically a cosmopolitan, saprophytic fungus which directly invades epidermal cell due to its pathogenicity. Therefore, the evolution of their pathogenicity in host plant pathogenic fungi is an important case for studying. *A. alternata* isolates infect host plants *via* directly penetrating fungal hyphae that form small infection peg grows that enters the host sensitive plant. *Alternaria* toxins are chemically similar to compounds that have biochemical effects as various cell organelles like, membrane leakage, inhibit protein synthesis, disrupt photo-phosphorylation, inhibit cell divisions, hormonal imbalance, and interferes plant metabolites activity. Definitely, in the future, the *Alternaria* toxins will be needed to explore for comprehensive analysis of their results, and it will expect, the significant analysis of the role of *Alternaria* toxins and their mode of action during pathogenesis against the plants. The key questions which will need to be answered in the near future are whether these toxins may be used as probes for rapid screening of plant clones or the progeny from crosses for the development of disease resistant varieties of plants. These toxins may act as antibiotic and could be involved in bio-control of noxious pathogens. It will be possible in the near future that these toxins may be used to develop disease free plants for future generations.

## Author contributions

MM conceived the idea of review, provided the general concept and inputs for each specific section, and drafted part of the manuscript. MM and SS wrote the manuscript. MM prepared all the figures and tables. Both the authors read and approved it for publication.

## References

[1] Thomma B.P.H.J. (2003). *Alternaria* spp.: from general saprophyte to specific parasite. Mol. Plant Pathol..

[2] Fernández R.L., Rivera M.C., Varsallona B., Wright E.R. (2015). Disease prevalence and symptoms caused by *Alternaria tenuissima* and *Pestalotiopsis guepinii* on blueberry in Entre Ríos and Buenos Aires, Argentina. Am. J. Plant Sci..

[3] Lee H.B., Patriarca A., Magan N. (2015). *Alternaria* in food: ecophysiology, mycotoxin production and toxicology. Mycobiology.

[4] Meena M., Gupta S.K., Swapnil P., Zehra A., Dubey M.K., Upadhyay R.S. (2017). *Alternaria* toxins: potential virulence factors and genes related to pathogenesis. Front. Microbiol..

[5] Meena M., Swapnil P., Upadhyay R.S. (2017). Isolation, characterization and toxicological potential of tenuazonic acid, alternariol and alternariol monomethyl ether produced by *Alternaria* species phytopathogenic on plants. Sci. Rep..

[6] Meena M., Zehra A., Dubey M.K., Aamir M., Gupta V.K., Upadhyay R.S. (2016). Comparative evaluation of biochemical changes in tomato (*Lycopersicon esculentum* Mill.) infected by *Alternaria alternata* and its toxic metabolites (TeA, AOH, and AME). Front. Plant Sci..

[7] Dickens J.S.W., Cook R.T.A. (1995). Alternaria pear black spot and apple blotch. Bulletin. OEPP/EPPO Bulletin..

[8] Tanaka A., Shiotani H., Yamamoto M., Tsuge T. (1999). Insertional mutagenesis and cloning of the genes required for biosynthesis of the hostspecific AK-toxin in the Japanese pear pathotype of *Alternaria alternata*. Mol. Plant-Microbe Interact..

[9] Berestetskiy A.O. (2008). A review of fungal phytotoxins: from basic studies to practical use. Appl. Biochem. Microbiol..

[10] Friesen T.L., Faris J.D., Solomon P.S., Oliver R.P. (2008). Host-specific toxins: effectors of necrotrophic pathogenicity. Cell Microbiol..

[11] Meena M., Prasad V., Zehra A., Gupta V.K., Upadhyay R.S. (2015). Mannitol metabolism during pathogenic fungal–host interactions under stressed conditions. Front. Microbiol..

[12] Meena M., Zehra A., Dubey M.K., Upadhyay R.S. (2016). Mannitol and proline accumulation in *Lycopersicum esculentum* during infection of *Alternaria alternata* and its toxins. Int. J. Biomed. Sci. Bioinformatics.

[13] Yoder O.C. (1980). Toxins in pathogenesis. Annu. Rev. Phytopathol..

[14] Nishimura S., Kohmoto K. (1983). Host-specific toxins and chemical structures from *Alternaria* species. Annu. Rev. Phytopathol..

[15] Scheffer R.P., Livingston R.S. (1984). Host-selective toxins and their role in plant diseases. Science.

[16] Markham J.E., Hille J. (2001). Host-selective toxins as agents of cell death in plant-fungus interactions. Mol. Plant Pathol..

[17] Wolpert T.J., Dunkle L.D., Ciuffetti L.M. (2002). Host-selective toxins and avirulence determinants: what’s in a name?. Annu. Rev. Phytopathol..

[18] Howlett B.J. (2006). Secondary metabolite toxins and nutrition of plant pathogenic fungi. Curr. Opin. Plant Biol..

[19] Kohmoto K., Otani H., Tsuge T., Kohmoto K., Singh U.S., Singh R.P. (1995). *Alternaria alternata* pathogens.

[20] Otani H., Kohmoto K., Kodama M. (1995). *Alternaria* toxins and their effects on host plants. Can. J. Bot..

[21] Quayyum H.A., Gijzen M., Traquair J.A. (2003). Purification of a necrosisinducing, host-specific protein toxin from spore germination fluid of *Alternaria panax*. Phytopathology..

[22] Akimitsu K., Tsuge T., Kodama M. (2013). *Alternaria* host-selective toxins: determinant factors of plant disease. J. Gen. Plant Pathol..

[23] Mishra S., Singh A., Keswani C., Saxena A., Sarma B.K., Singh H.B., Arora N.K. (2015). Harnessing plant-microbe interactions for enhanced protection against phytopathogens. Plant Microbe Symbiosis – Applied Facets.

[24] Keswani C., Bisen K., Singh V., Sarma B.K., Singh H.B., Arora N.K. (2016). Formulation technology of biocontrol agents: present status and future prospects. Bioformulations: for Sustainable Agriculture.

[25] Keswani C., Bisen K., Singh S.P., Sarma B.K., Singh H.B., Hakeem K.R., Akhtar M.S. (2016). A proteomic approach to understand the tripartite interactions between plant-*Trichoderma*-pathogen: investigating the potential for efficient biological control. Plant, Soil and Microbes Vol. 2.Mechanisms and Molecular Interactions.

[26] Tsuge T., Harimotao Y., Akamatsu K., Ohtani K., Kodama M., Akagi Y. (2013). Host–selective toxins produced by the plant pathogenic fungus *Alternaria alternata*. FEMS Microbiol. Rev..

[27] Takaoka S., Kurata M., Harimoto Y., Hatta R., Yamamoto M., Akimitsu K. (2014). Complex regulation of secondary metabolism controlling pathogenicity in the phytopathogenic fungus *Alternaria alternata*. New Phytol..

[28] Adhikari P., Oh Y., Panthee D. (2017). Current status of early blight resistance in tomato: an Update. Int. J. Mol. Sci..

[29] Ostry V. (2008). *Alternaria* mycotoxins: an overview of chemical characterization, producers, toxicity, analysis and occurrence in foodstuffs. World Mycotoxin J..

[30] Alexander J., Benford D., Boobis A., Ceccatelli S., Cottrill B., Cravedi J., di Domenico A., Doerge D., Dogliotti E., Edler L. (2011). Scientific opinion on the risks for animal and public health related to the presence of *Alternaria* toxins in feed and food. EFSA J..

[31] Hickert S., Hermes L., Marques L.M., Focke C., Cramer B., Lopes N.P., Flett B., Humpf H.U. (2017). *Alternaria* toxins in South African sunflower seeds: cooperative study. Mycotoxin Res..

[32] Meena M., Prasad V., Upadhyay R.S. (2017). Evaluation of *Alternaria alternata* isolates for metabolite production isolated from different sites of Varanasi, India. J. Agric. Res..

[33] Meena M., Prasad V., Upadhyay R.S. (2017). Evaluation of biochemical changes in leaves of tomato infected with *Alternaria alternata* and its metabolites. Vegetos.

[34] Meena M., Zehra A., Swapnil P., Dubey M.K., Patel C.B., Upadhyay R.S. (2017). Effect on lycopene, β-carotene, ascorbic acid and phenolic content in tomato fruits infected by *Alternaria alternata* and its toxins (TeA, AOH and AME). Arch. Phytopathol. Plant Prot..

[35] EFSA Panel on Contaminants in the Food Chain (CONTAM) (2011). Scientific opinion on the risks for animal and public health related to the presence of *Alternaria* toxins in feed and food. EFSA J..

[36] Vlata Z., Porichis F., Tzanakakis G., Tsatsakis A., Krambovitis E. (2005). *In vitro* cytopathic effects of mycotoxin T-2 on human peripheral blood T lymphocytes. Toxicol. Lett..

[37] Vlata Z., Porichis F., Tzanakakis G., Tsatsakis A., Krambovitis E. (2006). A study of zearalenone cytotoxicity on human peripheral blood mononuclear cells. Toxicol. Lett..

[38] Janić Hajnal E., Mastilović J., Bagi F., Orčić D., Budakov D., Kos J., Savić Z. (2019). Effect of wheat milling process on the distribution of *Alternaria* toxins. Toxins.

[39] Rychlik M., Lepper H., Weidner C., Asam S. (2016). Risk evaluation of the *Alternaria* mycotoxin tenuazonic acid in foods for adults and infants and subsequent risk management. Food Control.

[40] Bottini A.T., Gilchrist D.G. (1981). Phytotoxins I. A 1-aminodimethylheptadecapentol from *Alternaria alternata* f. sp. *lycopersici*. Tetrahedron Lett..

[41] Kohmoto K., Itoh Y., Shimomura N., Kondoh Y., Otani H., Kodama M. (1993). Isolation and biological activities of two host-specific toxins from the tangerine pathotype of *Alternaria alternata*. Phytopathology.

[42] Abbas H.K., Tanaka T., Duke S.O., Porter J.K., Wray E.M., Hodges L. (1994). Fumonisin- and AAL-toxin induced disruption of sphingolipid metabolism with accumulation of free sphingoid bases. Plant Physiol..

[43] Nakatsuka S., Ueda K., Goto T., Yamamoto M., Nishimura S., Kohmoto K. (1986). Structure of AF-toxin II, one of the host-specific toxins produced by *Alternaria alternata* strawberry pathotype. Tetrahedron Lett..

[44] Lee H.B., Kim C.J., Yu S.H. (2001). First report of strawberry fruit rot caused by *Alternaria tenuissima* in Korea. Plant Dis..

[45] Nakashima T., Ueno T., Fukami H., Taga T., Masuda H., Osaki K. (1985). Isolation and structure of AK-toxin I and II, host specific phytotoxic metabolites produced by *Alternaria alternata* Japanese pear pathotype. Agric. Biol. Chem..

[46] Izumi Y., Ohtani K., Miyamoto Y., Masunaka A., Fukumoto T., Gomi K. (2012). A polyketide synthase gene, *ACRTS2*, is responsible for biosynthesis of host-selective ACR-toxin in the rough lemon pathotype of *Alternaria alternata*. Mol. Plant-Microbe Interact..

[47] Gardner J.M., Kono Y., Tatum J.H., Suzuki Y., Takeuchi S. (1985). Structure of the major component of ACRL-toxins, host-specific pathotoxic compound produced by *Alternaria citri*. Agric. Biol. Chem..

[48] Akimitsu K., Kohmoto K., Otani H., Nishimura S. (1989). Host-specific effect of toxin from the rough lemon pathotype of *Alternaria alternata* on mitochondria. Plant Physiol..

[49] Kohmoto K., Otani H., Nishimura S., Nishimura S. (1987). Primary action sites for hostspecific toxins produced by *Alternaria* species. Molecular Determinants of Plant Diseases.

[50] Ueno T. (1990). Secondary metabolites related to host selection by plant pathogenic fungi. Pure Appl. Chem..

[51] Bains P.S., Tewari J.P. (1987). Purification, chemical charecterization and host-specficty of the toxin produced by *Alternaria brassicae*. Physiol. Mol. Plant Pathol..

[52] Stierle A.C., Cardellina J.H., Strobel G.A. (1988). Maculosin, a host-specific phytotoxin for spotted knapweed from *Alternaria alternata*. Proc. Natl. Acad. Sci. U. S. A..

[53] Stierle A.C., Cardellina I.I., Strobel G.A. (1989). Phytotoxins from *Alternaria alternata*, a pathogen of spotted knapweed. J. Nat. Prod..

[54] Pedras M.S.C., Zaharia I.L., Gai Y., Smith K.C., Ward D.E. (1999). Metabolism of the host-selective toxins destruxin B and homodestruxin B: probing a plant disease resistance trait. Org. Lett..

[55] Liakopoulou-Kyriakides M., Lagopodi A.L., Thanassoulopoulos C.C., Stavropoulos G.S., Magafa V. (1997). Isolation and synthesis of a host-selective toxin produced by *Alternaria alternata*. Phytochemistry.

[57] Ma Y.-T., Qiao L.-R., Shi W.-Q., Zhang A.-L., Gao J.-M. (2010). Metabolites produced by an endophyte *Alternaria alternata* isolated from *Maytenus hookeri*. Chem. Nat. Compd..

[58] Todd R.B., Andrianopoulos A. (1997). Evolution of a fungal regulatory gene family: the Zn(II)Cys6 binuclear cluster DNA binding motif. Fungal Genet. Biol..

[59] Chaube H.S., Pundhir V.S. (2005). Crop Diseases and Their Management.

[60] Taj G., Meena P.D., Giri P., Pandey D., Kumar A., Kumar A. (2015). Pathogenesis mechanisms employed by *Alternaria* species. J. Oilseed Brassica.

[61] Gardner J.M., Kono Y., Tatum J.H., Suzuki Y., Takeuchi S. (1985). Plant pathotoxins from *Alternaria citri*: the major toxin specific for rough lemon plants. Phytochemistry.

[62] Kono Y., Gardner J.M., Suzuki Y., Takeuchi S. (1985). Plant pathotoxins from *Alternaria citri*: the minor ACRL toxins. Phytochemistry.

[63] Kohmoto K., Kondo Y., Kohguchi T., Otani H., Nishimura S., Scheffer R.P. (1984). Ultrastructural changes in host leaf cells caused by host-selective toxin of *Alternaria alternata* from rough lemon. Can. J. Bot..

[64] Masunaka A., Ohtani K., Peever T.L., Timmer L.W., Tsuge T., Yamamoto M. (2005). An isolate that is pathogenic to both tangerines and rough lemon and produces two host-selective toxins, ACT- and ACR-toxins. Phytopathology.

[65] Ohtani K., Yamamoto H., Akimitsu K. (2002). Sensitivity to *Alternaria alternata* toxin in citrus because of altered mitochondrial RNA processing. Proc. Natl. Acad. Sci. U. S. A..

[67] Caldas E.D., Jones A.D., Ward B., Winter C.K., Gilchrist D.G. (1994). Structural characterization of three new AAL-toxins produced by *Alternaria alternata* f. sp. *lycopersici*. J. Agric. Food Chem..

[68] Spassieva S.D., Markham J.E., Hille J. (2002). The plant disease resistance gene Asc-1 prevents disruption of sphingolipid metabolism during AAL-toxin-induced programmed cell death. Plant J..

[69] Gilchrist D.G. (1998). Programmed cell death in plant disease: the purpose and promise of cellular suicide. Annu. Rev. Phytopathol..

[70] Hannun Y.A., Luberto C. (2000). Ceramide in the eukaryotic stress response. Trends Cell Biol..

[71] Liang H., Yao N., Song J.T., Luo S., Lu H., Greenberg J.T. (2003). Ceramides modulate programmed cell death in plants. Genes Dev..

[72] Wang H., Li J., Bostock R.M., Gilchrist D.G. (1996). Apoptosis: a functional paradigm for programmed plant cell death induced by a host-selective phytotoxin and invoked during development. Plant Cell.

[73] Moore T., Martineau B., Bostock R.M., Lincoln J.E., Gilchrist D.G. (1999). Molecular and genetic characterization of ethylene involvement in mycotoxin-induced plant cell death. Physiol. Mol. Plant Pathol..

[74] Mase K., Mizuno T., Ishihama N., Fujii T., Mori H., Kodama M. (2012). Ethylene signaling pathway and MAPK cascades are required for AAL toxin-induced programmed cell death. Mol. Plant-Microbe Interact..

[75] Gechev T., Gadjev I., Hille J. (2004). An extensive microarray analysis of AAL-toxin-induced cell death in *Arabidopsis thaliana* brings new insights into the complexity of programmed cell death in plants. Cell. Mol. Life Sci..

[76] Brandwagt B.F., Mesbah L.A., Takken F.L.W., Lauren P.L., Kneppers T.J.A., Hille J. (2000). A longevity assurance gene homolog of tomato mediates resistance to *Alternaria alternata* f. sp. *lycopersici* toxins and fumonisin B-1. Proc. Natl. Acad. Sci. U. S. A..

[77] Prasad V., Upadhyay R.S. (2010). *Alternaria alternata* f. sp. *lycopersici* and its toxin trigger production of H_2_O_2_ and ethylene in tomato. J. Plant Pathol..

[78] Gechev T.S., Ferwerda M.A., Mehterov N., Laloi C., Qureshi M.K., Hille J. (2008). *Arabidopsis* AAL toxin-resistant mutant atr1 shows enhanced tolerance to programmed cell death induced by reactive oxygen species. Biochem. Biophys. Res. Commun..

[79] Wang E., Norred W.P., Bacon C.W., Riley R.T., Merrill A.H. (1991). Inhibition of sphingolipid biosynthesis by fumonisins. Implications for diseases associated with *Fusarium moniliforme*. J. Biol. Chem..

[80] Lynch D.V., Merril A.H., Hannun Y.A. (1999). Enzymes of sphingolipid metabolism in plants. Sphingolipid Metabolism.

[81] Ismaiel A.A., Papenbrock J. (2015). Mycotoxins: producing fungi and mechanisms of phytotoxicity. Agriculture.

[82] Zhang L., Jia C., Liu L., Li C., Wang Q. (2011). Involvement of jasmonates and ethylene in *Alternaria alternata* f. sp. *lycopersici* toxin-induced tomato cell death. J. Exp. Bot..

[83] Akamatsu H., Itoh Y., Kodama M., Otani H., Kohmoto K. (1997). AAL-toxin deficient mutants of *Alternaria alternata* tomato pathotype by restriction enzyme-mediated integration. Phytopathology.

[84] Akamatsu H., Otani H., Kodama M. (2003). Characterization of a gene cluster for host-specific AAL-toxin biosynthesis in the tomato pathotype of *Alternaria alternata*. Fungal Genet. Newsl..

[85] Zhu X., Vogeler C., Du L. (2008). Functional complementation of fumonisin biosynthesis in FUM1 -disrupted *Fusarium verticillioides* by the AAL-toxin polyketide synthase gene *ALT1* from *Alternaria alternata* f. sp. *lycopersici*. J. Nat. Prod..

[86] Desjardins A.E., Munkvold G.P., Plattner R.D., Proctor R.H. (2002). FUM1—a gene required for fumonisin biosynthesis but not for maize ear rot and ear infection by *Gibberella moniliformis* in field tests. Phytopathology.

[87] Kuroyanagi M., Yamada K., Hatsugai N., Kondo M., Nishimura M., Hara-Nishimura I. (2005). Vacuolar processing enzyme is essential for mycotoxin-induced cell death in Arabidopsis thaliana. J. Biol. Chem..

[88] Desjardins A.E., Busman M., Proctor R.H., Stessman R. (2007). Wheat kernel black point and fumonisin contamination by *Fusarium proliferatum*. Food Addit. Contam..

[89] Ramires F., Masiello M., Somma S., Villani A., Susca A., Logrieco A., Luz C., Meca G., Moretti A. (2018). Phylogeny and mycotoxin characterization of *Alternaria* species isolated from wheat grown in Tuscany, Italy. Toxins..

[90] Abbas H.K., Duke S.O., Paul R.N., Riley R.T., AAL-toxin Tanaka T. (1995). A potent natural herbicide which disrupts sphingolipid metabolism of plants. Pest Manag. Sci..

[91] Ueno T., Nakashima T., Hayashi Y., Fukami H. (1975). Structures of AM-toxin I and II host-specific phytotoxic metabolites produced by *Alternaria mali*. Agric. Biol. Chem..

[92] Miyashita M., Nakamori T., Murai T., Miyagawa H., Akamatsu M., Ueno T. (1998). Facile syntheses of AM -toxins and analogs as cyclic depsipeptides by the solid-phase method. Biosci. Biotechnol. Biochem. (Japan).

[93] Park P., Nishimura S., Kohmoto K., Otani H., Tsujimoto K. (1981). Two action sites of AM-toxin I produced by apple pathotype of *Alternaria alternata* in host cell: an ultrastructural study. Can. J. Bot..

[94] Kohmoto K., Otani H., Nishimura S., Asada Y., Bushnell W.R., Ouchi S., Vance C.P. (1982). Action sites of AM-toxins produced by the apple pathotype of *Alternaria alternata*. Plant Infection: The Physiologocal and Biochemical Basis.

[95] Keller N.P., Turner G., Bennett J.W. (2005). Fungal secondary metabolism–biochemistry to genomics. Nat. Rev. Microbiol..

[96] Harimoto T., Hatta R., Kodama M., Yamamoto M., Otani H., Tsuge T. (2007). Expression profiles of genes encoded by the supernumerary chromosome controlling AM-toxin biosynthesis and pathogenicity in the apple pathotype of *Alternaria alternata*. Mol. Plant-Microbe Interact..

[97] Tsuge T., Harimoto Y., Hanada K., Akagi Y., Kodama M., Akimitsu (2016). Evolution of pathogenicity controlled by small, dispensable chromosomes in *Alternaria alternata* pathogens. Physiol. Mol. Plant Pathol..

[98] Park P., Ikeda K. (2008). Ultrastructural analysis of responses of host and fungal cells during plant infection. J. Gen. Plant Pathol..

[99] Kohmoto K., Otani H. (1991). Host recognition by toxigenic plant pathogens. Experientia.

[100] Kodama M., Ogata T., Sakamoto S., Sato S., Honda T., Miwatani T. (1990). Production of paralytic shellfish toxins by a bacterium *Moraxella* sp. isolated from *Protogonyaulax tamarensis*. Toxicon.

[101] Martin S.J., Green D.R. (1995). Protease activation during apoptosis: death by a thousand cuts?. Cell..

[102] Yakimova E.T., Yordanova Z.P., Slavov S., Kapchina-Toteva V.M., Woltering E.J. (2009). *Alternaria alternata* AT toxin induces programmed cell death in tobacco. J. Plant Pathol..

[103] Yamamoto M., Namiki F., Nishimura S., Kohmoto K. (1985). Studies on host-specific toxins produced by *Alternaria alternata* strawberry pathotype causing Alternaria black spot of strawberry (3) Use of toxin for determining inheritance of disease reaction in strawberry cultivar Morioka-16. Ann. Phytopathol. Soc. Jpn..

[104] Nishimura S., Nakatsuka S., Kohmoto K., Durbin R.D. (1989). Trends in host-selective toxin research in Japan. Host-Specific Toxins: Recognition and Specificity Factors in Plant Disease.

[105] Maekawa N., Yamamoto M., Nishimura S., Kohmoto K., Kuwada K., Watanabe Y. (1984). Studies on host-specific AF-toxins produced by *Alternaria alternata* strawberry pathotype causing Alternaria black spot of strawberry. (1) Production of host-specific toxins and their biological activities. Ann. Phytopathol. Soc. Jpn..

[106] Hatta R., Shinjo A., Ruswandi S., Kitani K., Yamamoto M., Akimitsu K. (2006). DNA transposon fossils present on the conditionally dispensable chromosome controlling AF-toxin biosynthesis and pathogenicity of *Alternaria alternata*. J. Gen. Plant Pathol..

[107] Otani H., Kohmoto K., Nishimura S., Nakashima T., Ueno T., Fukami H. (1985). Biological activities of AK-toxins I and II, host-specific toxins from *Alternaria alternata* Japanese pear pathotype. Ann. Phytopathol. Soc. Jpn..

[108] Foyer C.H., Noctor G. (2003). Redox sensing and signalling associated with reactive oxygen in chloroplasts, peroxisomes and mitochondria. Physiol. Plant..

[109] Tanaka A., Tsuge T. (2000). Structural and functional complexity of the genomic region controlling AK-toxin biosynthesis and pathogenicity in the Japanese pear pathotype of *Alternaria alternata*. Mol. Plant-Microbe Interact..

[110] Wight W.D., Labuda R., Walton J.D. (2013). Conservation of the genes for HC-toxin biosynthesis in *Alternaria jesenskae*. BMC Microbiol..

[111] Imazaki A., Tanaka A., Harimoto Y., Yamamoto M., Akimitsu K., Park P. (2010). Contribution of peroxisomes to secondary metabolism and pathogenicity in the fungal plant pathogen *Alternaria alternata*. Eukaryot. Cell.

[112] Uemura I., Miyagawa H., Ueno T. (2002). Asymmeteric total synthesis of AK-toxins. Tetrahedron Lett..

[113] Bobylev M.M., Bobyleva L.I., Strobel G.A. (1996). Synthesis and bioactivity of analogs of maculosin, a host-specific phytotoxin produced by *Alternaria alternata* on spotted knapweed (*Centaurea maculosa*). J. Agric. Food Chem..

[114] Park S.H., Stierle A., Strobel G.A. (1993). Metabolism of maculosin, a host-specific phytotoxin produced by *Alternaria alternata* on spotted knapweed (*Centaurea maculosa*). Phytochemistry.

[115] Sakamura S., Natori A., Ikekawa N., Suzuki M. (1981). Phytotoxins produced by plant pathogenic fungi. Advances in Natural Products Chemistry.

[116] Suzuki A., Taguchi H., Tamura S. (1970). Isolation and structure elucidation of three new insecticidal cyclodepsipeptides, destruxins C and D and desmethyldestruxin B, produced by *Metarhizium anisopliae*. Agric. Biol. Chem..

[117] Ayer W.A., Peña-Rodriguez L.M. (1987). Metabolites produced by *Alternaria brassicae*, the black spot pathogen of canola. Part 1, the phytotoxic components. J. Nat. Prod..

[118] Buchwaldt L., Green H. (1992). Phytotoxicity of destruxin B and its possible role in the pathogenesis of *Alternaria brassicae*. Plant Pathol..

[119] Montemurro N., Visconti A., Chełkowski J., Visconti A. (1992). *Alternaria* metabolites –Chemical and biological data. *Alternaria*: Biology, Plant Diseases and Metabolites.

[120] Pais M., Das B.C., Ferron P. (1981). Depsipeptides from *Metarhizium anisopliae*. Phytochemistry.

[121] Agarwal A., Garg G.K., Sing U.S., Mishra D.P. (1994). Detection and role of chlorotic toxin and phytohormones in the pathogenesis of Alternaria blight in *Brassica napus*. Curr. Sci..

[122] Tewari J.P., Bains P.S., Rajeev K., Mukerji K.G. (1997). Phytotoxins produced by *Alternaria brassicae* and bioassay of destruxin B. Toxins in Plant Disease Development and Evolving Biotechnology.

[123] Parada R.Y., Oka K., Yamagish D., Kodama M., Otani H. (2007). Destruxin B produced by *Alternaria brassicae* does not induce accessibility of host plants to fungal invasion. Physiol. Mol. Plant Pathol..

[124] Otani H., Kohnobe A., Kodama M., Kohmoto K. (1998). Production of a host-specific toxin by germinating spores of *Alternaria brassicicola*. Physiol. Mol. Plant Pathol..

[125] Oka K., Akamatsu H., Kodama M., Nakajima H., Kawada T., Otani H. (2005). Host-specific AB-toxin production by germinating spores of *Alternaria brassicicola* is induced by a host-derived oligosaccharide. Physiol. Mol. Plant Pathol..

[126] Otani H., Kohmoto K., Chelkowski J., Visconti A. (1992). Host-specific toxins of *Alternaria* species. *Alternaria*: Biology, Plant Diseases and Metabolites.

[128] Namiki F., Okamoto H., Katou K., Yamamoto M., Nishimura S., Nakatsuka S., Goto T., Kohmoto K., Otani H., Novacky A. (1986). Studies on host-specific AF-toxins produced by *Alternaria alternata* strawberry pathotype causing Alternaria black spot of strawberry (V). Effect of toxins on membrane potential of susceptible plants as assessed by electrophysiological method. Ann. Phytopathol. Soc. Jpn..

[129] Otani H., Tomiyama K., Okamoto H., Nishimura S., Kohmoto K. (1989). Effect of AK-toxin produced by *Alternaria alternata* Japanese pear pathotype on membrane potential of pear cells. Ann. Phytopathol. Soc. Jpn..

[130] Park P., Lee S.S., Ohno T., Tsuge T., Nishimura S. (1992). Slightly damaged type of plasma membrane modification in strawberry leaves treated with AF-toxin I produced by *Alternaria alternata* strawberry pathotype. Ann. Phytopathol. Soc. Jpn..

[131] Pedras M.S.C., Zaharia I.L., Ward D.E. (2002). The destruxins: synthesis, biosynthesis, biotransformation, and biological activity. Phytochemistry.

[132] Meena M., Swapnil P., Zehra A., Dubey M.K., Aamir M., Patel C.B., Upadhyay R.S., Singh H.B., Gupta V.K., Jogaiah S. (2018). Virulence factors and their associated genes in microbes. New and Future Developments in Microbial Biotechnology and Bioengineering.

[133] Meena M., Aamir M., Vikas K., Swapnil P., Upadhyay R.S. (2018). Evaluation of morpho-physiological growth parameters of tomato in response to Cd induced toxicity and characterization of metal sensitive NRAMP3 transporter protein. Environ. Exp. Bot..

[134] Pedras M.S.C., Zaharia I.L., Gai Y., Zhou Y., Ward D.E. (2001). In planta sequential hydroxylation and glycosylation of a fungal phytotoxin: avoiding cell death and overcoming the fungal invader. Proc. Natl. Acad. Sci. U. S. A..

[135] Multani D.S., Meeley R.B., Paterson A.H., Gray J., Briggs S.P., Johal G.S. (1998). Plant-pathogen microevolution: molecular basis for the origin of a fungal disease in maize. Proc. Natl. Acad. Sci. U. S. A..

[136] Andersen B., Nielsen K.F., Pinto V.F., Patriarca A. (2015). Characterization of *Alternaria* strains from Argentinean blueberry, tomato, walnut and wheat. Int. J. Food Microbiol..

[137] Vlata Z., Tsatsakis A., Tzagournissakis M., Krambovitis E. (2012). Evaluation of specific immune responses to BoNT/A and tetanus toxoid in patients undergoing treatment for neurologic disorders. Endocr. Metab. Immune Disord. Drug Targ. (Formerly Current Drug Targets-Immune, Endocrine & Metabolic Disorders)..

[138] Wu W., Zhou H.R., Pan X., Pestka J.J. (2015). Comparison of anorectic potencies of the trichothecenes T-2 toxin, HT-2 toxin and satratoxin G to the ipecac alkaloid emetine. Toxicol. Rep..

[139] Logrieco A., Moretti A., Solfrizzo M. (2009). *Alternaria* toxins and plant diseases: an overview of origin, occurrence and risks. World Mycotoxin J..

[140] Bessadat N., Berruyer R., Hamon B., Bataille-Simoneau N., Benichou S., Kihal M., Henni D.E., Simoneau P. (2017). *Alternaria* species associated with early blight epidemics on tomato and other *Solanaceae* crops in northwestern Algeria. Eur. J. Plant Pathol..

[141] Yekeler H., Bitmiş K., Ozçelik N., Doymaz M.Z., Calta M.E. (2001). Analysis of toxic effects of *Alternaria* toxins on esophagus of mice by light and electron microscopy. Toxicol. Pathol..

[142] Solhaug A., Vines L.L., Ivanova L., Spilsberg B., Holme J.A., Pestka J., Collins A., Eriksen G.S. (2012). Mechanisms involved in alternariol-induced cell cycle arrest. Mutat. Res. Fundam. Mol. Mech. Mutagen..

[143] Lehmann L., Wagner J., Metzler M. (2006). Estrogenic and clastogenic potential of the mycotoxin alternariol in cultured mammalian cells. Food Chem. Toxicol..

[144] Solhaug A., Wisbech C., Christoffersen T.E., Hult L.O., Lea T., Eriksen G.S., Holme J.A. (2015). The mycotoxin alternariol induces DNA damage and modify macrophage phenotype and inflammatory responses. Toxicol. Lett..

[145] Brugger E.M., Wagner J., Schumacher D.M., Koch K., Podlech J., Metzler M., Lehmann L. (2006). Mutagenicity of the mycotoxin alternariol in cultured mammalian cells. Toxicol. Lett..

[146] Pfeiffer E., Eschbach S., Metzler M. (2007). *Alternaria* toxins: DNA strand-breaking activity in mammalian cells *in vitro*. Mycotoxin Res..

[147] Pahlke G., Tiessen C., Domnanich K., Kahle N., Groh I.A., Schreck I., Weiss C., Marko D. (2016). Impact of *Alternaria* toxins on CYP1A1 expression in different human tumor cells and relevance for genotoxicity. Toxicol. Lett..

[148] Fleck S.C., Burkhardt B., Pfeiffer E., Metzler M. (2012). *Alternaria* toxins: altertoxin II is a much stronger mutagen and DNA strand breaking mycotoxin than alternariol and its methyl ether in cultured mammalian cells. Toxicol. Lett..

[149] Asam S., Lichtenegger M., Muzik K., Liu Y., Frank O., Hofmann T., Rychlik M. (2013). Development of analytical methods for the determination of tenuazonic acid analogues in food commodities. J. Chromatogr. A.

[150] Stockmann-Juvala H., Savolainen K. (2008). A review of the toxic effects and mechanisms of action of fumonisin B1. Hum. Exp. Toxicol..

[151] Cota B.B., Rosa L.H., Caligiorne R.B., Rabello A.L., Almeida Alves T.M., Rosa C.A., Zani C.L. (2008). Altenusin, a biphenyl isolated from the endophytic fungus *Alternaria* sp., inhibits trypanothione reductase from *Trypanosoma cruzi*. FEMS Microbiol. Lett..

[152] Johann S., Rosa L.H., Rosa C.A., Perez P., Cisalpino P.S., Zani C.L., Cota B.B. (2012). Antifungal activity of altenusin isolated from the endophytic fungus *Alternaria* sp. against the pathogenic fungus *Paracoccidioides brasiliensis*. Revista iberoamericana de Micologia.

[153] Mendoza M. (2011). Oxidative burst in plant-pathogen interaction. Biotecnología Vegetal..

[154] Grant J.J., Yun B.W., Loake G.J. (2000). Oxidative burst and cognate redox signalling reported by luciferase imaging:identification of a signal network that functions independently of ethylene, SA and Me-JA but is dependent on MAPKK activity. Plant J..

[155] Liu Y., Ren D., Pike S., Pallardy S., Gassmann W., Zhang S. (2007). Chloroplast-generated reactive oxygen species are involved in hypersensitive response-like cell death mediated by amitogen-activated protein kinase cascade. Plant J..

[156] Mittler R., Herr E.H., Orvar B.L., van Camp W., Willekens H., Inzé D. (1999). Transgenic tobacco plants with reduced capability to detoxify reactive oxygen intermediates are hyper-responsive to pathogen infection. Proc. Natl. Acad. Sci. U. S. A..

[157] Klessig D.F., Durner J., Noad R., Navarre D.A., Wendehenne D., Khou J.M. (2000). Nitric oxide and salicylic acid signaling in plant defense. Proc. Natl. Acad. Sci. U. S. A..

[158] Sharma P., Jha A.B., Dubey R.S., Pessarakli M. (2012). Reactive oxygen species, oxidative damage, and antioxidative defense mechanism in plants under stressful conditions. J. Bot..

[159] Torres M.A., Jones J.D.G., Dangl J.L. (2006). Reactive oxygen species signaling in response to pathogens. Plant Physiol..

[160] Shetty N.P., Lyngs Jørgensen H.J., Due Jensen J., Collinge D.B., Shekar Shetty H. (2008). Roles of reactive oxygen species in interactions between plants and pathogens. Eur. J. Plant Pathol..

[161] Das K., Roychoudhury A. (2014). Reactive oxygen species (ROS) and response of antioxidants as ROS-scavengers during environmental stress in plants. Front. Environ. Sci..

[162] Meena M., Zehra A. (2019). Tomato: a model plant to study plant-pathogen interactions. Food Sci. Nutr. Technol..

[163] Miller E.W., Dickinson B.C., Chang C.J. (2010). Aquaporin-3 mediates hydrogen peroxide uptake to regulate downstream intracellular signalling. PNAS..

[164] Heller J., Tudzynski P. (2011). Reactive oxygen species in phytopathogenic fungi: signaling, development, and disease. Annu. Rev. Phytopathol..

[165] Meena M., Prasad V., Upadhyay R.S. (2016). Assessment of the bioweedicidal effects of *Alternaria alternata* metabolites against *Parthenium* species. Bull. Environ. Sci. Res..

[166] Meena M., Swapnil P., Zehra A., Dubey M.K., Upadhyay R.S. (2017). Antagonistic assessment of *Trichoderma* spp. by producing volatile and non-volatile compounds against different fungal pathogens. Arch. Phytopathol. Plant Prot..

[167] Meena M., Swapnil P. (2019). Regulation of *WRKY* genes in plant defense with beneficial fungus *Trichoderma*: current perspectives and future prospects. Arch. Phytopathol. Plant Prot..

[168] Shetty N.P., Kristensen B.K., Newman M.A., Møller K., Gregersen P.L., Jørgensen H.J.L. (2003). Association of hydrogen peroxide with restriction of *Septoria tritici* in resistant wheat. Physiol. Mol. Plant Pathol..

[169] Suzuki N., Miller G., Morales J., Shulaev V., Torres M.A., Mittler R. (2011). Respiratory burst oxidases: the engines of ROS signalling. Curr. Opin. Plant Biol..

[170] Nathan C., Cunningham-Bussel A. (2013). Beyond oxidative stress: an immunologist’s guide to reactive oxygen species. Nat. Rev. Immunol..

[171] Gilroy S., Suzuki N., Miller G., Choi W.G., Toyota M., Devireddy A.R. (2014). A tidal wave of signals: calcium and ROS at the forefront of rapid systemic signalling. Trends Plant Sci..

[172] Ram R.M., Keswani C., Bisen K., Tripathi R., Singh S.P., Singh H.B., Brah D., Azevedo V. (2018). Biocontrol technology: eco-friendly approaches for sustainable agriculture. Omics Technologies and Bio-Engineering: Towards Improving Quality of Life Volume II Microbial, Plant, Environmental and Industrial Technologies.

[173] Singh H.B., Sarma B.K., Keswani C. (2016). Agriculturally Important Microorganisms: Commercialization and Regulatory Requirements in Asia.

[174] Singh H.B., Sarma B.K., Keswani C. (2017). Advances in PGPR Research.

[175] Singh H.B., Keswani C., Reddy M.S., Royano E.S., García-Estrada C. (2019). Secondary Metabolites of Plant Growth Promoting Rhizomicroorganisms: Discovery and Applications.

[176] Wrzaczek M., Brosche M., Kangasjarvi J. (2013). ROS signaling loops – production, perception, regulation. Curr. Opin. Plant Biol..

[177] Singh H.B., Jain A., Saxena A., Singh A., Keswani C., Sarma B.K., Mishra S., Sharma N. (2014). Deciphering the pathogenic behaviour of phytopathogens using molecular tools. Biological Controls for Preventing Food Deterioration: Strategies for Pre- and Postharvest Management.

[178] Singh V.K., Meena M., Zehra A., Tiwari A., Dubey M.K., Upadhyay R.S., Kharwar R.N., Upadhyay R.S., Dubey N.K., Raghuwanshi R. (2014). Fungal toxins and their impact on living systems. Microbial Diversity and Biotechnology in Food Security.

[179] Patel C.B., Singh V.K., Singh A.P., Meena M., Upadhyay R.S., Singh H.B., Gupta V.K., Jogaiah S. (2018). Microbial genes involved in interaction with plants. New and Future Developments in Microbial Biotechnology and Bioengineering.

[180] Apel K., Hirt H. (2004). Reactive oxygen species: metabolism, oxidative stress, and signal transduction. Annu. Rev. Plant Biol..

